# The influence of the theory and methods of Chinese traditional culture on modern western psychotherapy: a cross-cultural dialogue

**DOI:** 10.3389/fpsyg.2026.1690911

**Published:** 2026-04-13

**Authors:** Danqing Yin, Fang Xu

**Affiliations:** 1 Li Ka Shing Faculty of Medicine, School of Biomedical Sciences, The University of Hong Kong, Hong Kong, Hong Kong SAR, China; 2 School of Social Development, Nanjing Normal University, Nanjing, Jiangsu, China

**Keywords:** Buddhism, Chinese philosophical psychology, Confucianism, cross-cultural psychotherapy, Daoism, mind healing, perception

## Introduction

Historically, before the origination of modern psychotherapy, those in distress turn to temples and Daoist sanctuaries to seek relief from their worries and perplexities. Monks and priests, through their teachings and guidance, often fulfilled the role that today we would recognize as akin to a counselor or psychotherapist.

As one of the world's four ancient civilizations, China has cultivated a long-standing tradition of mental health care. The philosophies of Confucianism, Buddhism, and Daoism have been especially effective in easing distress and suffering, offering indigenous approaches to psychological wellbeing. In today's context of cultural confidence and the national “Healthy China” strategy, recovering and advancing the traditional principles and methods of *tiao xin* (调心)—the regulation and harmonization of the mind—holds great significance for addressing the mental health needs of modern society.

### Theories and models of *Tiao Xin* (调心, regulation of mind) in Chinese traditions

Among the traditions of Confucianism, Buddhism, and Daoism, it is Buddhism that most directly addresses the afflictions of the mind. According to Buddhist philosophy (Liu, 2019; Sun, 2019), human beings perceive the phenomenal world—*se, sheng, xiang, wei, chu, fa* (色、声、香、味、触、法: form, sound, fragrance, taste, touch, and mental objects)—through the six faculties of*yan, er, bi, she, shen, yi* (眼、耳、鼻、舌、身、意: eye, ear, nose, tongue, body, and mind). Because of *wu ming* (无明, ignorance; *avidyā*), people misapprehend these phenomena and develop dualistic judgments—existence vs. non-existence, good vs. evil, beauty vs. ugliness. Clinging to such discriminations (*fen bie xin*, 分别心, discriminative mind) gives rise to *shou, xiang, xing, shi* (受、想、行、识: sensation, perception, volition, and consciousness), which in turn engender *fan nao* (烦恼, *klesha*, mental afflictions) and suffering.

In this framework (Zhu, 2009), the Buddhist account of how ordinary beings fall into distress can be mapped as: **phenomena (**色**, se)** **→ sensory faculties (**六根**, six sense bases)** **→** **discriminative mind (**分别心**, fen** **bie xin)** **→ clinging to self (*wo zhi***, 我执**;** **ā*tma-gr*ā*ha*)** **→** **afflictions (*fan nao***, 烦恼**)**.

If reframed in the language of modern psychology—echoing Jung's account of the sequence from perception to judgment—the process can be expressed as: **material phenomena**→**perception**→**evaluation**→**emotions and desires**→**distress**.

Since *fan nao* (mental afflictions) arise through the dynamic interplay of self and environment, the traditions of Confucianism, Buddhism, and Daoism all offer methods of *tiao xin* (调心, regulation of mind) that intervene at different stages:

- **Cultivating heightened perception** (*qiang diao gan zhi*, 强调感知), training the senses to encounter the world more directly.
- **Practicing non-discrimination** (*bu fen bie*, 不分别; *nirvikalpa-jñāna*), suspending habitual dualistic judgments.
- **Regulating emotions and desires** (*guanli qinggan yuyuwang*, 管理情感与欲望), akin to emotion regulation in modern psychology.
- **Turning inward for self-cultivation** (*xiang nei qiu*, 向内求; inner contemplation), seeking the source of wisdom within rather than outside.

**Figure 1:**
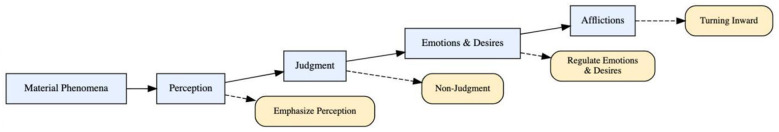
Conceptual model of tiao xin (调心, regulation of mind) in Chinese traditional culture.

A conceptual model of tiao xin (调心, regulation of mind) in Chinese traditional culture can be derived from abovementioned framework, as illustrated in Figure 1.

### Emphasis on perception in Confucianism, Buddhism, and Daoism, and its influence on western psychotherapy

#### Methods of emphasizing perception in Confucianism, Buddhism, and Daoism

Within the traditions of Confucianism, Buddhism, and Daoism—most explicitly in Buddhism—in which practices featuring perception (*gan zhi*,感知) begin with stillness. All three traditions advocate **sitting meditation (*jing zuo***, 静坐**)** as a foundational discipline.

- In Confucianism, *jing zuo* mediates “regulation of body” (*tiao shen*, 调身), aligning posture and comportment with moral enrichment.
- In Buddhism, *jing zuo* functioned to “regulate the mind” (*tiao xin*, 调心), calming and stabilizing awareness. Related practices included *guan chan* (观禅, insight meditation), *guan hu xi* (观呼吸, mindfulness of breathing), *guan nian tou* (观念头, observing thoughts), and *bu xing chan* (步行禅, walking meditation).
- In Daoism, *jing zuo* was directed toward health cultivation (*yang sheng*, 养生), nourishing inner vitality and harmony with the Dao.

Together, these practices fostered refined sensory awareness and mindful presence as a way of attuning body and mind.

Based on long-term practical experience, the traditions of Confucianism, Buddhism, and Daoism recognized that discursive thinking (si wei huo dong, 思维活动) generates afflictions (fan nao, 烦恼) and obstructs the realization of the Way (wu dao, 悟道). A gongan (public case) recorded in Jingde Chuandeng Lu (“景德传灯录”) recounts that Chan Master ZhiXian (Zhi Xian chan shi, 智闲禅师) in his early years extensively studied sutras under Master Baizhang but failed to awaken to the Chan mystery. Feeling that his intellectual pursuit of words was like “drawing a cake to satisfy hunger” (hua bing chong ji, 画饼充饥) and of no benefit to the Way, he burned all his collected writings. One day, by chance, he tossed a tile fragment that struck a bamboo stalk, producing a clear, sharp sound. Suddenly, he experienced great enlightenment (da wu, 大悟). He then composed a verse: “With one strike, all that was known is forgotten; no need for further cultivation.” This story illustrates how the sound of the tile striking bamboo shattered the constraints of linguistic concepts, leading to instantaneous realization. It elucidates Chan Buddhism's emphasis on perception and its doctrine of sudden enlightenment (dun wu, 顿悟), which requires no deliberate cultivation.

To regulate the mind (tiao xin, 调心), all three traditions value perception (gan zhi, 感知). A key practice method for enhancing perception is sitting meditation (jing zuo, 静坐).

In Confucianism, jing zuo mediates the “regulation of the body” (tiao shen, 调身). Mencius (Mengzi, 孟子), Xunzi (Xunzi, 荀子), and Zhu Xi (Zhu Xi, 朱熹) all practiced regular sitting meditation.

In Buddhism, jing zuo functions to “regulate the mind” (tiao xin, 调心). Sitting meditation (da zuo, 打坐) is the most commonly used Buddhist practice. The Heart Sutra (Xin Jing, “心经”) opens with a scene of sitting meditation: “Avalokiteshvara Bodhisattva, when practicing deeply the Prajnaparamita, perceives that all five aggregates are empty, and is saved from all suffering and distress.” Related practices include insight meditation (guan chan, 观禅), mindfulness of breathing (guan hu xi, 观呼吸), observing thoughts (guan nian tou, 观念头), and walking meditation (bu xing chan, 步行禅).

In Daoism, jing zuo is primarily a method for “nourishing life” (yang sheng, 养生). The Daodejing (“道德经”) states: “Attain ultimate emptiness; abide firmly in stillness.” The Zhuangzi (“庄子”) contains concepts such as “sitting in forgetfulness” (zuo wang, 坐忘) and “fasting of the mind” (xin zhai, 心斋). According to the Zhuangzi, the Yellow Emperor once sought the secret of longevity from a sage named Guang Chengzi (Guang Chengzi, 广成子), who replied: “Look at nothing, listen to nothing; keep your spirit still and your body will correct itself. Be still, be pure; do not weary your body, do not disturb your vitality, and you will live long. When the eyes see nothing, the ears hear nothing, and the mind knows nothing, your spirit will guard your body, and your body will endure forever.”

Because perception is immediate, all three traditions emphasize dwelling in the present moment (dang xia, 当下s). A well-known Chan Buddhist gongan illustrates this: “Better go drink tea.” Two monks arrived at Zhaozhou's monastery. Master Congshen (Cong Shen chan shi, 从谂禅师) asked one, “Have you been here before?” The monk replied, “Yes.” The master said, “Go drink tea” (chi cha qu, 吃茶去). He then asked the other monk, who replied, “No.” The master also said, “Go drink tea.” Later, the monastery's steward asked the master, “Why did you tell both monks—one who had been here and one who had not—to go drink tea?” The master replied, “Go drink tea.” The meaning is clear: let go of thoughts about past and future, return to the present moment, and simply drink tea.

#### The influence of emphasizing perception on western psychotherapy

In terms of awareness, modern Gestalt therapy posits that the brain's functions can be divided into three zones: (1) the outer zone, which involves seeing, hearing, smelling, and tasting through our sensory organs such as the eyes, ears, and nose; (2) the middle zone, referring to the cerebral cortex, which governs cognitive activities such as perception, judgment, and logic; and (3) the inner zone, which refers to brain regions responsible for emotional experiences and bodily responses, such as the limbic system.

Modern people tend to overemphasize the analytical function of the brain (the middle zone) while neglecting sensation (the outer zone) and emotion (the inner zone). This imbalance in utilizing the three brain zones causes excessive energy to be concentrated in thinking, leading to wandering thoughts and unnecessary afflictions. As a result, people eat without truly tasting their food, are mentally elsewhere while physically present, and experience a disconnection between body and mind. Therefore, modern individuals need to readjust the focus of brain energy and strengthen perception.

##### Living in the present moment

Judgment (pan duan, 判断) is an analytical thinking activity of the cerebral cortex. When excessive energy is consumed in this area, individuals become unable to experience the present moment (dang xia, 当下), leading to afflictions (fan nao, 烦恼).

In Buddhism, particular emphasis is placed on awareness of the present moment. A similar stance characterizes **Gestalt therapy**, which regards its central feature as the focus on “the here and now.” Gestalt therapy holds that modern individuals often fail to live in the present, neglecting the immediacy of perception. Instead, they cling to memories of the past or fantasies of the future, which in effect serve as an avoidance of present-moment experience. In both Buddhist practice and Gestalt therapy, cultivating awareness of “this very moment” is considered essential—believing that learning to observe, know, and feel the fullness of the present as the primary gateway to healing.

##### Valuing sensation

Gestalt therapy also resonates with the Buddhist emphasis on *awareness* (*jue zhi*, 觉知), highlighting the balance of different modes of brain function. In this framework, brain activity can be divided into three domains:

- **Outer brain region**—sensory inputs received through the eyes, ears, nose, tongue, and skin.
- **Middle brain region**—the cerebral cortex, responsible for cognition, evaluation, and logical reasoning.
- **Inner brain region**—areas governing emotional experience and somatic response, such as the limbic system.

Modern life, Gestalt theorists suggest, has led to an over-reliance on the middle brain's analytic function, while neglecting sensory perception (outer brain) and emotional attunement (inner brain). This imbalance concentrates excessive energy on discursive thinking, generating restless thoughts (*wang nian*, 妄念) and thereby increasing *fan nao* (烦恼, mental afflictions). As a result, one may lose the ability to taste food fully, find the body present but the mind elsewhere, and experience a split between body and mind. To restore balance, modern individuals must re-adjust their emphasis, learning once again to value sensation and embodied presence.

### Non-judgment in Confucianism, Buddhism, and Daoism, and its influence on western psychotherapy

#### Methods of non-judgment in Confucianism, Buddhism, and Daoism

In matters of judgment, Confucianism advocates the *Doctrine of the Mean* (*zhongyong*, 中庸). The *Shang Shu* (“尚书·大禹谟”) states: “The human heart is perilous, the Dao-heart subtle; be refined, unified, and hold faithfully to the Mean (允执厥中).” Here, *yun zhi jue zhong* (允执厥中) signifies speaking and acting without deviation, embodying a balanced, impartial way.

In Buddhism, *fen bie xin* (分别心, the discriminative mind) is identified as a root cause of *fan nao* (烦恼, klesha, mental afflictions). To counteract this, Buddhism teaches the *gate of non-duality* (*bu erfa men*, 不二法门), which dissolves clinging to dualistic distinctions such as existence and non-existence. In Chan (Zen) practice, this is expressed through methods such as *can hua tou* (参话头, meditative inquiry into a koan), *ji feng* (机锋, sharp repartee), and the use of shouts and blows (*bang he*, 棒喝) to break through conceptual fixation.

Daoism, for its part, presents the doctrine of *Qi Wu Lun* (齐物论, “Equalizing Things”), expressed in the words of Zhuangzi: “Heaven and I are born together; the 10 thousand things and I are one.” This contains both *qi wu* (齐物)—the view that the multiplicity of things is ultimately one—and *qi lun* (齐论)—the recognition that differing perspectives and judgments are, in essence, without absolute right or wrong.

#### The influence of non-judgment on western psychotherapy

##### Cognitive change and its impact on judgment

Similar to Buddhism's critique of *wang nian* (妄念, deluded thoughts) and *fen bie xin* (discriminative cognition), Western rational emotive behavior therapy (REBT) and Beck's cognitive therapy hold that the source of emotional and behavioral problems lies not in external events themselves, but in people's interpretations of those events. Cognition—one's recognition and appraisal of reality—serves as the mediator between event and affliction.

Distorted, irrational, or rigid elements in cognition (*cognitive distortions*, such as negative automatic thoughts and dysfunctional schemas) are core contributors to emotional disturbance and psychological suffering. By modifying maladaptive cognitions, negative emotions and behaviors can be reduced. The Buddhist aim of overcoming *fen bie xin* parallels the goals of cognitive therapy: transforming one's interpretation of events, breaking through distorted cognition, dissolving *wang nian*, and ultimately achieving *ming xin jian xing* (明心见性, illuminating the mind and seeing one's true nature)—though the methods differ.

##### Recognizing the subjectivity of judgment

Postmodern psychology likewise challenges the notion of objective judgment. It posits that the world is not an independent reality but a subjective construction, that knowledge is co-created through dialogue rather than bestowed by authority, and that language is a cultural product rather than a vessel of eternal truth. Narrative therapy, a prominent postmodern approach, employs the questioning of “objective judgment” as a therapeutic tool. It argues that many standards, beliefs, values, expectations, and conventions—taken for granted as “judgments”—are socially constructed discourses, produced through cultural context and interpersonal interaction. These discourses regulate people's thoughts and behaviors, shaping them into “healthy” or “normal” persons, and thus reflect mechanisms of social power and control. Yet such beliefs and customs are not immutable truths; they evolve with historical change. For example, attitudes toward out-of-wedlock pregnancy have shifted significantly across time.

##### The body–mind–spirit unity

In the late 1960s, transpersonal psychology, emerging from the foundations of humanistic psychology, drew profound influence from Buddhism and meditative practices. It sought to integrate the wisdom of global spiritual traditions into modern psychology. Central to this movement is the view that the human being is a unity of body, mind, and spirit. Psychological problems, therefore, cannot be confined to the somatic or cognitive domains alone, but also involve the dimension of spirit—phenomena not yet fully understood within modern science but essential to a holistic understanding of human suffering and transformation.

### Methods of regulating emotions and desires in Confucianism, Buddhism, and Daoism, and their influence on modern western psychotherapy

#### Methods of regulating emotions and desires

Confucian society places a high value on collective values, emphasizing “face” (mian zi, 面子), prestige, and a rigidly hierarchical family and social order based on Confucian ethics. It imposes strict requirements on the regulation of emotions and desires, as exemplified by the injunction to “preserve heavenly principle and eliminate human desires” (cun tian li, mie ren yu, 存天理、灭人欲). This entails opposition to free romantic choice, the promotion of arranged marriage (fu mu zhi ming, mei shuo zhi yan, 父母之命,媒妁之言), and the advocacy of chastity and widowhood as moral virtues. Due to the excessively repressive nature of Confucian orthodoxy (li xue, 儒家理学) in suppressing human nature, this may generate feelings of shame, depression, and even narcissistic personality disorders, while simultaneously discouraging individuals from seeking professional help.

In Buddhism, ignorance (*wu ming*, 无明; *avidyā*) is understood as the ultimate root of emotions and desires. The stronger one's *wo zhi* (我执, clinging to self; ā*tma-grāha*), the greater the suffering, expressed through the “three poisons”: *tan, chen, chi* (贪、嗔、痴; greed, anger, and delusion). Ordinary beings are driven by sensual desire and suffer deeply as a result. The Buddhist path prescribes the discipline of renunciation as a way to manage emotions and desires. This is embodied in the system of precepts (*jie*, 戒): the Five Precepts (*wu jie*, 五戒)—not killing, not stealing, abstaining from sexual misconduct, not lying, and not consuming intoxicants; and the Eight Precepts (*ba jie*, 八戒), which add refraining from high luxurious beds, adornments and entertainments, and eating after noon. Beyond these are the 250 rules for monks (*bi qiu jie*, 比丘戒) and 348 rules for nuns (*bi qiu ni jie*, 比丘尼戒), forming a comprehensive system of ethical regulation.

Daoism also advocates moderation of desire, emphasizing simplicity and restraint. Expressions such as *jian su bao pu* (见素抱朴, “see the simple, embrace the uncarved block”) and *shao si gua yu* (少私寡欲, “less selfishness, fewer desires”) highlight the Daoist ethic of returning to naturalness. The *Dao De Jing* cautions: “The five colors blind the eye; the five tones deafen the ear; the five flavors dull the palate. Pursuit of hunting leads the heart astray; rare goods hinder conduct. Thus the sage attends to the belly, not the eye, and chooses the one over the other.” This passage underscores the principle that unbridled pursuit of sensory stimulation leads to disorder, whereas moderation preserves harmony.

#### Influence on modern western psychotherapy

##### The use of sensation in regulating emotions and desires

Parallels can be drawn between Buddhist contemplative practices such as the white-bone *contemplation* (*bai gu guan*, 白骨观), as one of the five methods of Chan meditation in Buddhism, typically refers to the contemplation of impurity (bu jing guan, 不净观). It involves visualizing a corpse decaying until only white bones remain. The primary purpose is to extinguish attachment to the physical body and to break free from attachment to the self (wo zhi, 我执). It sees the body's impermanence to counter sensual desire, and behavioral therapy techniques like **aversion therapy** and **flooding therapy**. Aversion therapy applies aversive stimuli to extinguish maladaptive behaviors, while flooding therapy exposes patients to anxiety-provoking stimuli or situations, real or imagined, until the anxiety is diminished. Both approaches seek to transform the emotional power of desire through confronting its sensory basis.

Both Eastern and Western therapeutic approaches can achieve similar effects. Aversion therapy and flooding therapy primarily target behavioral modification, whereas the Buddhist contemplation of impurity (bu jing guan, 不净观), operating at a philosophical level, facilitates a more fundamental transformation of both cognition and behavior. Although the contemplation of the white skeleton (bai gu guan, 白骨观) possesses therapeutic qualities, its application in modern secular therapeutic settings requires caution to prevent potential side effects such as depression and world-weariness.

##### The importance of sensation

Gestalt therapy emphasizes the social and experiential dimensions of emotions and desires. It posits that many emotional drives are shaped by “unfinished business”—unexpressed emotions such as regret, anger, resentment, pain, anxiety, grief, guilt, or feelings of abandonment. Because these emotions were not fully experienced within the perceptual field, they remain in the unconscious and intrude into present life without awareness. This unresolved affective residue distorts perception and fuels maladaptive desires, leading to psychological distress. Restoring balance thus requires re-engaging sensation, completing unfinished experiences, and reintegrating fragmented aspects of the self into embodied presence.

### Methods of addressing *Fan Nao* (烦恼, mental afflictions) in Confucianism, Buddhism, and Daoism, and their influence on modern western psychotherapy

#### Methods of addressing Fan Nao (烦恼, afflictions) in Confucianism, Buddhism, and Daoism

##### Accepting *Fan Nao*

In their attitudes toward *fan nao* (烦恼, mental afflictions), Confucianism tends toward a rational stance of rejection, while Daoism and Buddhism emphasize acceptance. However, while the Buddhist belief in “karmic retribution” (yin guo bao ying, 因果报应) or fatalism in Western context, can help individuals accept their current circumstances, it may also lead them to attribute their suffering to punishment for past transgressions. For example, Xianglin's Wife (Xianglin Sao, 祥林嫂), a character from Lu Xun's New Year's Sacrifice (Zhu Fu, “祝福”), mistakenly believed that her tragic fate was a result of karmic retribution and sought to atone by “donating a threshold” (juan men kan, 捐门槛). When working with clients, modern therapists should first clarify their clients' value orientations. Since clients may hold different Confucian, Buddhist, or Daoist value inclinations, their value backgrounds vary, and accordingly, therapeutic strategies should be tailored differently.

In Buddhist practice, acceptance is closely tied to the principle of mindfulness (*zheng nian*, 正念), which entails “non-judgmental acceptance.” On the one hand, this means suspending evaluative judgment; on the other, it means allowing both pleasant and painful experiences to arise without attempting to alter, control, or avoid them. This approach amounts to accepting afflictions as part of the lived experience.

##### Turning inward (*Xiang Nei Qiu*, 向内求)

Regarding who is the agent of working with afflictions, Confucianism places greater emphasis on external conditions, often attributing problems to the environment or social context. By contrast, Daoism and Buddhism emphasize *xiang nei qiu* (向内求, turning inward). Daoism advocates *sui yuan* (随缘, going with conditions), encouraging the individual to adapt flexibly to the external world. Buddhism teaches that beings, bound by ignorance since beginningless time, become lost in attachment and fail to recognize their true nature. Thus, the central task of practice is to rediscover this inherent nature (*jian xing cheng fo*, 见性成佛, seeing one's nature and becoming Buddha)—in other words, awakening. This entails turning inward to seek wisdom within, rather than projecting outward.

A clinical illustration can be an individual divorced with depressive mood seeking psychotherapist's help. These individual serves as a classic example of not practicing “turning inward” (*xiang nei qiu*, 向内求). He/she believed that happiness could only be achieved through external salvation, waiting for another person for dependence and rescue him/her from misery. This reflects a psychological pattern in which one's sense of worth is placed in the hands of others, rather than cultivated from within. If such an individual can come to realize that himself/herself is the master of his/her own life by realizing “turning inward,” he/she can begin to transform her behavior, shifting from passive waiting to active self-authorship. In contrast, a introspective Western approach psychotherapist might ask the suffered individual to reflect the root causes of this divorce, like patterns from family of origin repeated in his/her marriage.

#### Influence on modern western psychotherapy

##### Mobilizing inner resources

In resonance with Buddhism's advocacy of *xiang nei qiu* (turning inward), Jungian analytical psychology sets self-realization and wholeness as its ultimate goal. This parallels the Buddhist notion of *fo xing* (佛性, Buddha-nature). Even Jung's concept of the “collective unconscious” bears resemblance to the Buddhist eighth *consciousness* (*a lai ye shi*, 阿赖耶识; ālaya-vijñāna).

Similarly, Satir's family therapy also emphasizes turning inward and connecting with oneself. Satir argued that human beings share common universal yearnings: to be loved, accepted, recognized, affirmed; to possess safety, meaning, purpose, and freedom; to experience connection and belonging. To fulfill these, one must reconnect with one's own inner resources rather than depend solely on external supports. The self is intrinsically energetic and capable, yet people often focus on their shortcomings while overlooking their strengths. True help arises when individuals can rely on their own capacities.

##### Accepting *Fan Nao*

Satir's therapy also resonates with the Buddhist principle of acceptance, teaching that one must accept negative emotions as valid experiences because they serve as inner messengers. For example:

- Fear signals a lack of trust in oneself, others, and the universe.
- Anger indicates the inappropriate surrender of one's power.
- Fatigue suggests the need for more effective and intelligent ways of acting, inviting us to listen to our inner wisdom.
- Hurt offers the opportunity to practice forgiveness, which allows the self to be released into freedom.

Gestalt therapy also emphasizes the necessity of acknowledging unpleasant emotions. It observes that in order to avoid discomfort, people often resist contact and build defensive mechanisms, thereby evading the full, authentic experience of the present. This hinders genuine expression. Gestalt further stresses that pleasant and unpleasant emotions are relative and mutually transforming; thus the correct attitude is to acknowledge both, and to prepare to accept the unpleasant as part of wholeness.

Dialectical behavior therapy (DBT), integrating dialectical philosophy and Zen, warns that an overemphasis on change may heighten arousal and provoke resistance to therapy. Instead, DBT underscores the balance between “change” and “acceptance.”

In contemporary American psychology, Steven Hayes' Acceptance and Commitment Therapy (ACT) has carried this principle further. ACT emphasizes embracing suffering, recognizing that “happiness is not the norm of life,” and then building and enacting one's values within that reality.

Although both Chinese and Western therapies value “acceptance,” the concept differs in its fundamental orientation. In Buddhism, acceptance is a path toward enlightenment (jue wu, 觉悟), at a philosophical level. In contrast, acceptance in Western psychotherapy functions primarily as a tool for behavioral change.

## Conclusion

In summary, the *tiao xin* (调心, regulation of mind) theories and methods of Confucianism, Buddhism, and Daoism can be regarded as a form of **philosophical psychotherapy**. Their aim is to transform a person's philosophy of life—leading to shifts in behavior and emotion—through insight and realization. The principle underlying the generation of *fan nao* (烦恼, klesha, mental afflictions) may be rendered as: **material phenomena**→**perception**→**judgment**→**emotions and desires**→**afflictions**.

From the Buddhist perspective, four key therapeutic stages can be identified: **emphasizing perception, suspending judgment, regulating emotions and desires, and addressing afflictions**. These approaches, embedded in China's long intellectual and spiritual history, have deeply inspired and facilitated the development of modern psychotherapy.

Schools such as analytical psychology, Gestalt therapy, mindfulness-based therapies, dialectical behavior therapy (DBT), transpersonal psychology, and Acceptance and Commitment Therapy (ACT) all bear the imprint of Buddhist psychology and its methods.

Within the present context of cultural confidence and the national strategy of “Healthy China,” the task of rediscovering and revitalizing the principles and practices of *tiao xin* is of great significance. These traditions not only enrich the global dialogue between philosophy and psychotherapy, but also offer living resources for addressing the pressing mental health challenges of contemporary society.

On one hand, unlike traditional Chinese collectivist values, the West emphasizes individualist values. China and the West have different traditional cultural value backgrounds. Culturally specific indigenous Chinese psychological intervention theories and measures possess their own epistemological foundations and unique therapeutic mechanisms. The two exist independently and engage in equal dialogue. Even when similar concepts appear—for example, Buddhism's “turning inward” (xiang nei qiu, 向内求) means becoming one's own master, while Western “introspection” (nei xing, 内省), rooted in Christian confession, primarily involves reflection on one's own transgressions—they cannot be equated. On the other hand, based on shared underlying human psychological mechanisms, Chinese and Western psychotherapies have each independently developed into complete theoretical and practical systems, evolving convergently, while mutually influencing and promoting each other.
